# Troubleshooting the Trach: Emergent Tracheostomy & Laryngectomies Modified Team-Based Learning Activity

**DOI:** 10.21980/J8.52033

**Published:** 2025-10-31

**Authors:** Caroline Astemborski, Emily Grass

**Affiliations:** * Prisma Health Upstate, University of South Carolina School of Medicine Greenville, Department of Emergency Medicine, Greenville, SC; ^ University of Alabama Birmingham, Department of Emergency Medicine, Birmingham, AL

## Abstract

**Audience:**

Emergency medicine resident physicians (PGY1-4), medical students rotating in the emergency department

**Introduction:**

Tracheostomies are surgically created airways in the anterior of the neck to gain permanent or temporary access to the airway to facilitate oxygenation and ventilation.^1^ Tracheostomies are performed for management of upper airway obstruction, long-term ventilation, and airway protection.^1^ More than 110,000 tracheostomies are placed annually in the United States.^2^ Emergency Medicine Physicians need to recognize and treat tracheostomy complications because they can occur 40–50% of the time in patients with tracheostomies.^2^ Most tracheostomy complications are minor, but up to 1% are considered life-threatening complications that pose a significant morbidity and mortality.^2^ Laryngectomies are rarer but are typically performed for carcinoma of the larynx, disrupting the connection to the oropharynx.^1^ While tracheostomies and laryngectomies are found in a similar location, the management is vastly different due to changes in anatomy. This modified team-based learning activity will review anatomy, history, and common presentations of tracheostomy patients in the emergency department.

**Educational Objectives:**

By the end of the session, participants will be able to: identify the major anatomy of tracheostomies and laryngectomies, 2) demonstrate step-by-step management of emergent tracheostomy airways, 3) describe common complications of tracheostomies, 4) understand the management of tracheal innominate artery complication.

**Educational Methods:**

This team-based learning activity is a modified TBL that includes learner responsible content (LRC), a multiple-choice group readiness assessment test (gRAT) with immediate feedback assessment technique (IF/AT), and a group application exercise (GAE).

**Research Methods:**

A post-TBL survey was provided to each participant. A Likert scale was used for the survey questions to assess the relevance of the session to emergency medicine practice, learner perception of knowledge gained, learner perception of improvement of clinical practice, session engagement, and session delivery.

**Results:**

For this session, 26 participants completed the post-TBL survey. Participants included 3^rd^/4^th^ year medical students (19%), PGY1 (31%), PGY2 (23%), and PGY3 (27%) emergency medicine residents. Overall, 21/26 participants strongly agreed (5/5 Likert scale) and 4/26 agreed (4/5 Likert scale) that the session improved their knowledge of caring for tracheostomy patients with a weighted average of 4.78. Twenty-three participants (88.5%) strongly agreed (5/5 Likert scale) that the material in the TBL was relevant for practicing emergency medicine. The majority of participants (88.4%) found the session engaging. Narrative feedback included, “I am much more comfortable with trach complications.” Suggestions for improvement included, “would love to have time with a respiratory therapist to be available for questions during the session.” Narrative negative feedback included, “As a PGY-1, I haven’t had a lot of lessons on tracheostomies, so I felt a little useless during the application exercise.”

**Discussion:**

Airway emergencies for patients with tracheostomies and laryngectomies that are critical occur relatively infrequently. Given the rarity and high stakes of these events, it is important for emergency medicine trainees to have exposure to effective and high-yield learning activities which can aid in their preparedness to provide optimal care for patients presenting with tracheostomy-associated conditions.

This modified team-based learning (TBL) session facilitates discussion of pertinent anatomy and accurate equipment identification. Additionally, this session guides learners through a comprehensive step-by-step algorithmic approach to airway management for patients with a tracheostomy/laryngectomy presenting with obstruction, dislodgement, or tracheoinnominate fistula. Learners found the modified TBL (mTBL) to be beneficial for learning how to troubleshoot tracheostomy complications and manage these unique, challenging airway emergencies.

**Topics:**

Airway management, tracheostomies, laryngectomies, decannulation, obstruction.

## USER GUIDE

**Table:** 

List of Resources:	
Abstract	1
User Guide	3
Learner Materials	6
iRAT	6
gRAT	8
GAE	10
Instructor Materials	23
RAT Key	24
GAE Key	28
Debrief PowerPoint	41

**Learner Audience:**

Interns, Junior Residents, Senior Residents, Emergency Medicine Bound Medical Students

We recommend mixed learner groups. The topic is advanced, and novice learners may feel overwhelmed without mixed level of training.

**Time Required for Implementation:**

Instructor Preparation: 60–90 minutes

Learner Responsible Content: 30–45 minutes

In Class Time: 50 minutes

- Introduction: 5 minutes
- gRAT: 5–10 minutes
- GAE: 20–25 minutes
- Review: 10 minutes

**Recommended Number of Learners per Instructor:**

This modified TBL can be taught with one instructor, although it may be helpful to have an additional facilitator to circulate the room during the group application exercises to interact and guide the session. Additional facilitators could be chief or senior resident.

**Topics:**

Airway management, tracheostomies, laryngectomies, decannulation, obstruction.

**Objectives:**

By the end of the session, participants will be able to:

1. Identify the major anatomy of tracheostomies and laryngectomies.
2. Demonstrate step-by-step management of emergent tracheostomy airways.
3. Describe common complications of tracheostomies.
4. Understand the management of tracheal innominate artery fistula.

### Linked objectives and methods

Tracheostomy emergencies are uncommon but often involve complex and challenging presentations in the emergency department. The topic can be challenging for residents to practice due to the rarity of these high stakes presentations in the Emergency Department. A team-based learning activity provides an opportunity to navigate the complexities of these presentations, enabling learners to develop a structured algorithm for managing such cases. Group Activity Exercise #1 explores basics of tracheostomy equipment and anatomy related to tracheostomies and laryngectomies (Objective 1). Group Activity Exercise #2 examines a step-by-step pathway of the management of obstructive tracheostomy emergencies (Objectives 2 and 3). Group Activity Exercise #3 discusses definitive airway management (Objectives 1, 2, 3). Lastly, Group Activity Exercise #4 guides learners through management of a tracheal innominate artery fistula case. (Objectives 1, 2, 3, 4). The group application exercises build from basic anatomy questions to complex pathways on airway management, providing emergency medicine learners with an algorithm to manage these complex cases.

### Recommended pre-reading for instructor

- Bontempo LJ, Manning SL. Tracheostomy emergencies. *Emerg Med Clin North Am.* 2019;37(1):109–119. doi:10.1016/j.emc.2018.09.010
- McGrath BA, Bates L, Atkinson D, Moore JA. Multidisciplinary guidelines for the management of tracheostomy and laryngectomy airway emergencies. *Anaesthesia*. 2012;67(9):1025–1041. doi:10.1111/j.1365-2044.2012.07217.x
- Morgenstern J. Respiratory distress in the patient with a tracheostomy (update). First10EM. October 12, 2018. Accessed November 25, 2024. https://first10em.com/tracheostomy/

### Learner Responsible Content (LRC)

We recommend providing learners with a printout of the articles or attaching to a place where learners can easily access the resources. We have found that only providing the citation leads to difficulty with access and not all learners participating in the LRC.

- Bontempo LJ, Manning SL. Tracheostomy emergencies. *Emerg Med Clin North Am*. 2019;37(1):109–119. doi:10.1016/j.emc.2018.09.010
- McGrath BA, Bates L, Atkinson D, Moore JA. Multidisciplinary guidelines for the management of tracheostomy and laryngectomy airway emergencies. *Anaesthesia*. 2012;67(9):1025–1041. doi:10.1111/j.1365-2044.2012.07217.x

#### Optional Reading for Senior Residents

- Morgenstern J. Respiratory distress in the patient with a tracheostomy (update). First10EM. October 12, 2018. Accessed November 25, 2024. https://first10em.com/tracheostomy/

### Results and tips for successful implementation

#### Results

This modified TBL session was conducted in person in Summer 2023 at a tertiary academic center. The participants included emergency medicine residents post-graduate years 1–3 and emergency medicine-bound medical students. Groups consisted of five to seven learners at mixed level of training per group with a total of twenty-six participants. The session was evaluated with a post-activity evaluation using Likert scale questions and a space for narrative session feedback. Formative feedback from the faculty was that the activity was successful, and the TBL allowed for group interaction and critical thinking. For this session, 26 participants completed the post-TBL survey. Participants included 3^rd^/4^th^ year medical students (19%), PGY1 (31%), PGY2 (23%), and PGY3 (27%) emergency medicine residents. Overall, 21/26 participants strongly agreed (5/5 Likert scale) and 4/26 agreed (4/5 Likert scale) that the session improved their knowledge of caring for tracheostomy patients with a weighted average of 4.78. Twenty-three participants (88.5%) strongly agreed (5/5 Likert scale) that the material in the TBL was relevant for practicing emergency medicine. The majority of participants (88.4%) found the session engaging. Narrative feedback included, “I am much more comfortable with trach complications.” Suggestions for improvement included, “would love to have time with a respiratory therapist to be available for questions during the session.” Narrative negative feedback included, “As a PGY-1, I haven’t had a lot of lessons on tracheostomies, so I felt a little useless during the application exercise.”

#### Limitations & Recommendation for Future

Recommendations for future sessions include preassigning groups and allowing for more time for the GAE to allow for group discussion. We were constrained by time in our educational conference, but implementers could easily expand this mTBL to 60 or 90 minutes to allow for in-session LRC and expanded GAE discussion. Additionally, some of the new PGY1 residents did not complete the LRC prior to the session due to confusion of responsibilities. Lack of completing LRC resulted in frustration with the activity by some of the PGY1s. Recommendations for future would be to orient new learners to TBLs to ensure the understanding of pre-class requirements. We provided our learners the opportunity to explore trach equipment during the mTBL session. We would recommend having a few supplies for learners to see and explore in addition to completing the mTBL session. Limitations to the mTBL include single site study and the sample size of residents. Additionally, asking residents to evaluate their own educational activity could lead to undue influence on the results due to fear of providing negative feedback to program leadership.

#### Instructions for Implementation

Four weeks prior to the session, the instructor should:

1. Send out the learner responsible content to the learners.

Prior to the session, the instructor should prepare materials:

1. One gRAT per three to five learners per group
2. One GAE per three to five learners per group
3. Copies of all materials, including the keys for each instructor

You will need approximately 45 mins to conduct the session. We suggest the following timeline:

1. Introduce the session (5 mins)
  - The instructor assigns learners into groups of three to five. Ideally, groups will be a mix of learner levels.
2. Instructor hands out the gRAT to all groups of three to five learners. (5–10 mins)
3. Groups will complete the GAE. Faculty should circulate to be able to answer questions as appropriate. (20–25 mins)
4. Review answers from GAE using the supplemental Microsoft PowerPoint. (10 mins)
5. Hand out the GAE-Key as a post-activity review guide.

## Figures and Tables

**Figure:**
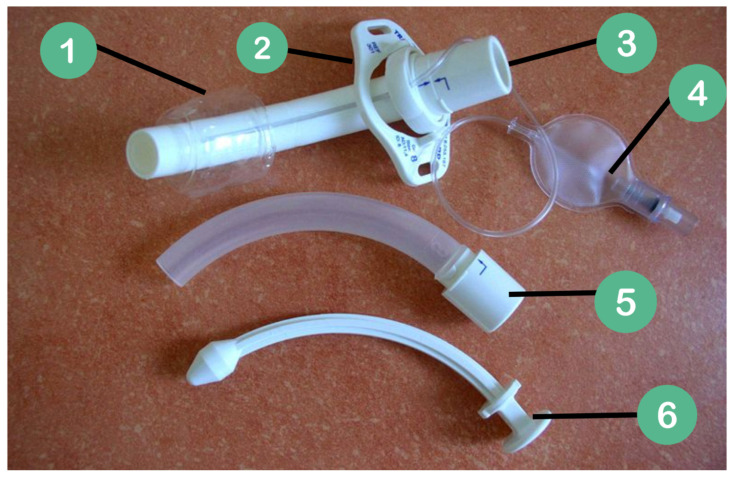
Klaus D. Peter, Wiehl, Germany. Tracheostomy. In: Wikimedia Commons. CC BY 3.0 DE
CuffFaceplateOuter CannulaPilot BalloonInner CannulaObturator

**Figure:**
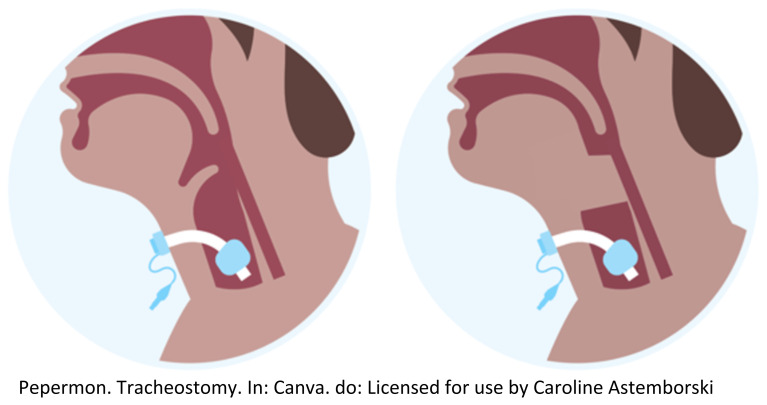
Pepermon. Tracheostomy. In: Canva. do: Licensed for use by Caroline Astemborski

**Figure:**
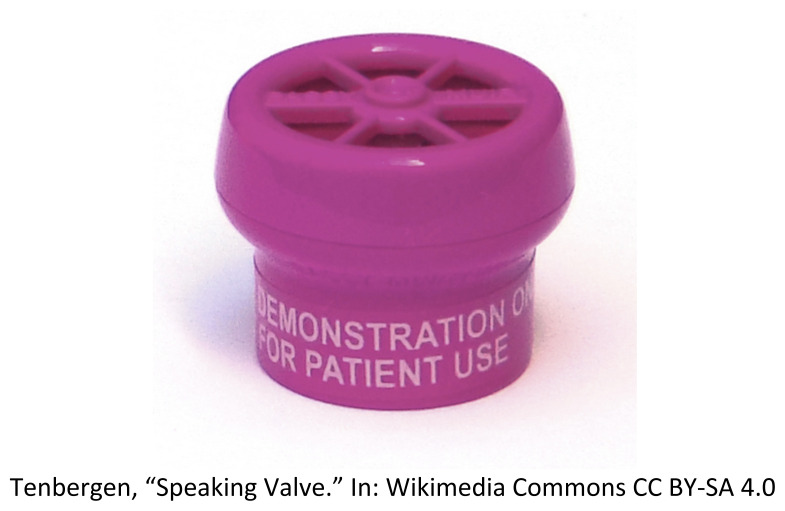
Tenbergen, “Speaking Valve.” In: Wikimedia Commons CC BY-SA 4.0

**Figure:**
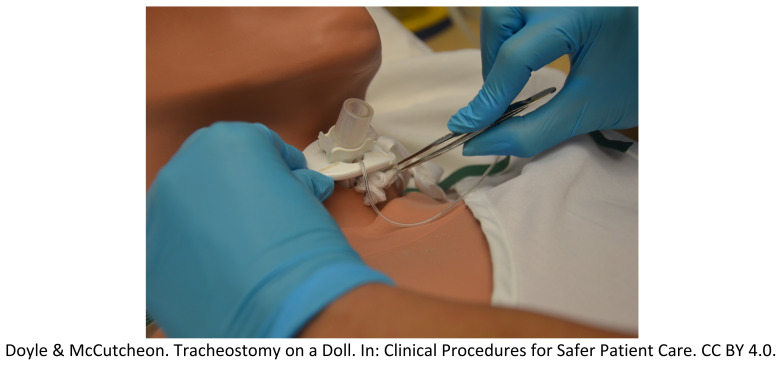
Doyle & McCutcheon. Tracheostomy on a Doll. In: Clinical Procedures for Safer Patient Care. CC BY 4.0.

**Figure:**
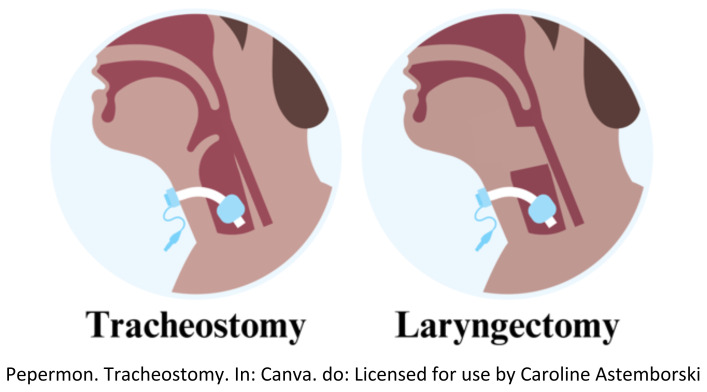
Pepermon. Tracheostomy. In: Canva. do: Licensed for use by Caroline Astemborski

**Figure:**
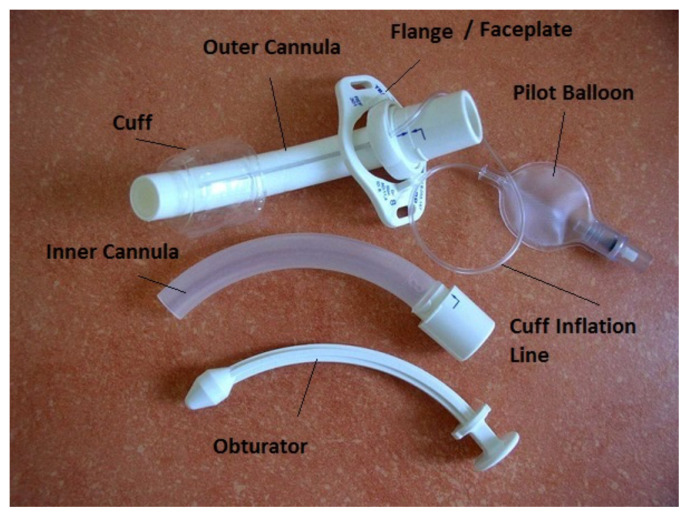
Peter K. Tracheostomy tube. In: Wikipedia. https://commons.wikimedia.org/wiki/File:Tracheostomy_tube.jpg. April 16 2008. CC BY 3.0.

**Figure:**
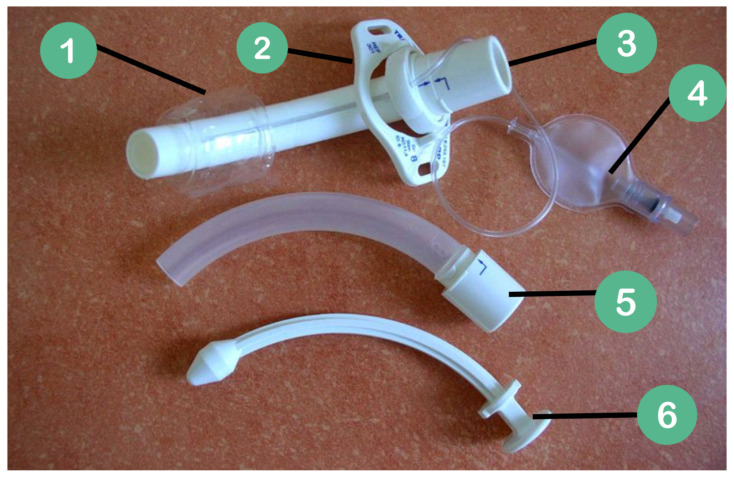
Klaus D. Peter, Wiehl, Germany. Tracheostomy. In: Wikimedia Commons. CC BY 3.0 DE
**Cuff****Faceplate****Outer Cannula****Pilot Balloon****Inner Cannula****Obturator**

**Figure:**
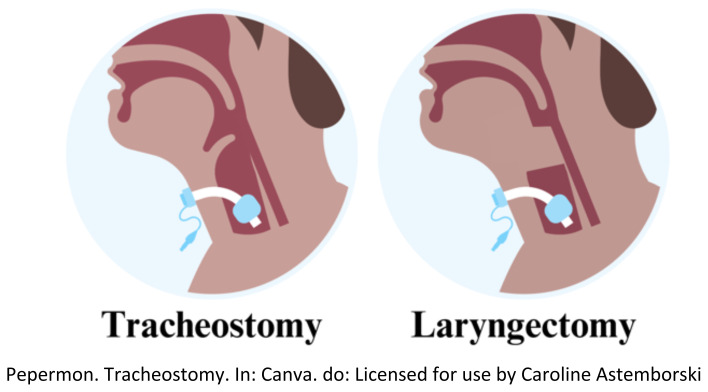
Pepermon. Tracheostomy. In: Canva. do: Licensed for use by Caroline Astemborski

**Figure:**
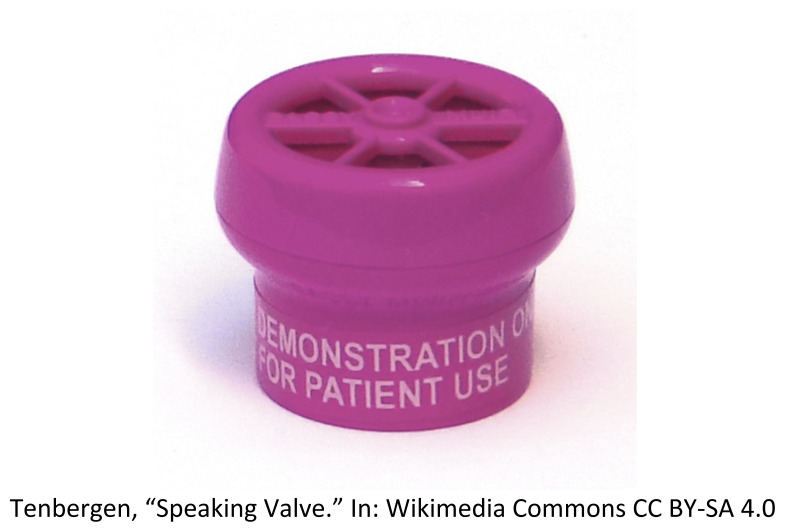
Tenbergen, “Speaking Valve.” In: Wikimedia Commons CC BY-SA 4.0

**Figure:**
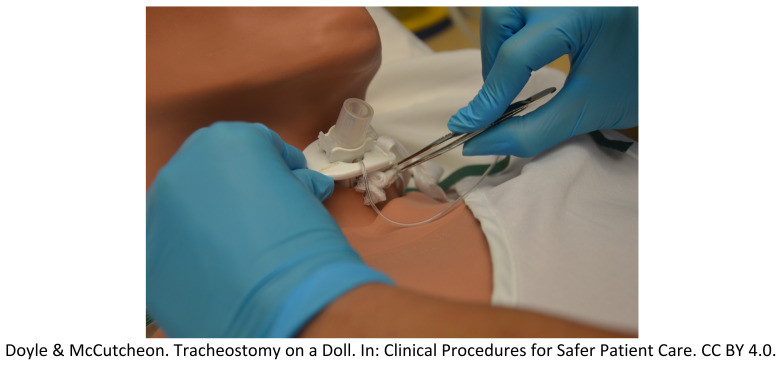
Doyle & McCutcheon. Tracheostomy on a Doll. In: Clinical Procedures for Safer Patient Care. CC BY 4.0.

**Figure:**
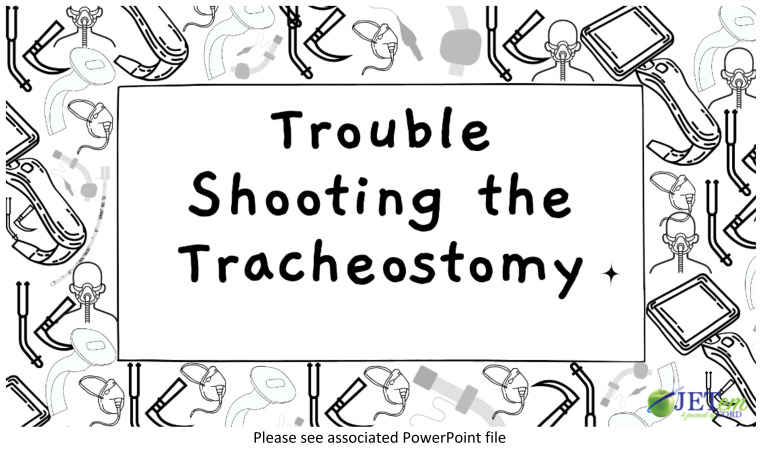
Please see associated PowerPoint file

## LEARNER MATERIALS

### Troubleshooting the Trach TBL: individual Readiness Assessment Test (iRAT)

1. Which of the following patients can be intubated from above?
  - Laryngectomy Patients
  - Laryngectomy and Tracheostomy Patients
  - Tracheostomy Patients
  - Neither Laryngectomy or Tracheostomy Patients
2. Which of the following is not a component of a tracheostomy tube?
  - Flange
  - Inner Cannula
  - Obturator
  - Outer Cannula
  - Regulator
3. At what postoperative day can a tracheostomy be safely exchanged via the stoma?
  - Post-op Day 1
  - Post-op Day 5
  - Post-op Day 6
  - Post-op Day 14
4. What is the second step in the algorithm for the treatment of a patient with an obstructed tracheostomy?
  - Attempt to pass a suction catheter
  - Deflate the cuff
  - Intubate from above.
  - Remove any speaking valve or external caps if present
  - Remove the inner cannula
5. A patient presents three days after discharge from prolonged hospital stay for respiratory failure requiring tracheostomy placement with bleeding from his tracheostomy site. His tracheostomy was placed 10 days ago. On inspection, he has a large amount of frank, bright red blood in his tracheostomy. His vital signs are BP 85/55, HR 125, Oxygen saturation 88% on room air, Temp 98.6° F, and RR 32. What is the first step in management of his condition?
  - Overinflate the tracheostomy cuff
  - Remove tracheostomy and intubate from above
  - Go directly to the operating room
  - Remove tracheostomy and apply direct pressure with digital manual compression anteriorly

### Troubleshooting the Trach TBL: group Readiness Assessment Test (gRAT)

1. Which of the following patients can be intubated from above?
  - Laryngectomy Patients
  - Laryngectomy and Tracheostomy Patients
  - ★ Tracheostomy Patients
  - Neither Laryngectomy or Tracheostomy Patients
2. Which of the following is not a component of a tracheostomy tube?
  - Flange
  - Inner Cannula
  - Obturator
  - Outer Cannula
  - ★ Regulator
3. At what postoperative day can a tracheostomy be safely exchanged via the stoma?
  - Post-op Day 1
  - Post-op Day 5
  - Post-op Day 6
  - ★ Post-op Day 14
4. What is the second step in the algorithm for the treatment of a patient with an obstructed tracheostomy?
  - Attempt to pass a suction catheter
  - Deflate the cuff
  - Intubate from above.
  - Remove any speaking valve or external caps if present
  - ★ Remove the inner cannula
5. A patient presents three days after discharge from prolonged hospital stay for respiratory failure requiring tracheostomy placement with bleeding from his tracheostomy site. His tracheostomy was placed 10 days ago. On inspection, he has a large amount of frank, bright red blood in his tracheostomy. His vital signs are BP 85/55, HR 125, Oxygen saturation 88% on room air, Temp 98.6° F, and RR 32. What is the first step in management of his condition?
  - ★ Overinflate the tracheostomy cuff
  - Remove tracheostomy and intubate from above
  - Go directly to the operating room
  - Remove tracheostomy and apply direct pressure with digital manual compression anteriorly

### Troubleshooting the Trach TBL: Group Application Exercise (GAE)

#### Group Application Exercise - Activity #1 – Tracheostomy Basics

1. Identify the parts of the tracheostomy:
2. Your patient needs to be ventilated. Which tracheostomy tube can you attach a bag-valve-mask (BVM) for ventilation?
3. What part of the tracheostomy tube do you attach to the BVM?
4. Your patient is unsure of their surgical history. Identify which patient had a tracheostomy and which had a laryngectomy.
5. Your patient is unsure of their type of tracheostomy tube. How can you tell the size of the tube? How can you tell if it is cuffed or uncuffed?
6. Your patient has a 6.0 cuffed Shley tube. You do not have this item stocked in your outlying hospital. What size endotracheal tube (ETT) could you place in the stoma?

#### Group Application Exercise - Activity #2 – Airway Algorithm

You are working in a rural emergency department (ED) as a single coverage physician when you receive a patient from a nearby skilled nursing facility for respiratory distress. The patient has a history of anoxic brain injury and has had a tracheostomy for many years. EMS state they were called by the nursing facility staff for low oxygen saturation and difficulty breathing.

Initial Vital Signs: HR 120 bpm, RR 36, BP 105/66, O2 72% on room air

They placed a non-rebreather mask on the patient and quickly transported him to you.

1. What is your first step in evaluating this patient?
2. What questions are important for you to ask this patient?

The patient has thready, rapid peripheral pulses. He is breathing spontaneously and appears to be in severe respiratory distress. His O2 on the monitor is 80% on NRB over the mouth. You assess his tracheostomy equipment and see a purple cap covering the inner cannula. You suspect airway obstruction.

3. What is the step that needs to be taken to relieve the airway obstruction?

Once you have removed the speaking cap, you examine the equipment below.

4. What is the next step in attempting to relieve airway obstruction? Hint: this step relieves the majority of airway obstructions.

The patient’s oxygen saturation improves minimally to 86%, but he still appears to have some increased work of breathing with coarse breath sounds and stridor.

5. Describe the steps of how to set up suctioning for a tracheostomy patient.

You attempt to perform suctioning of the patient but are unable to pass the suction catheter through the stoma. The patient remains hypoxic, tachypneic, and with stridor. You attempt to bag the patient via their tracheostomy tube; you are unable to get adequate tidal volumes and bagging is extremely difficult.

You follow the next step in the algorithm to aid the patient’s obstruction. You deflate the cuff and see if it helps the obstruction.

6. How can you determine how much air should be removed from the patient’s tracheostomy balloon to adequately deflate the cuff?

You deflate the cuff and the patient’s respiratory distress subsides. His oxygen saturation returns to normal. Your respiratory therapist assists with applying humidified oxygen and eventually you are able to suction out large amounts of mucus. He is found to have atelectasis on his chest radiograph likely from mucous plugging. You admit for observation.

#### Group Application Exercise - Activity #3 - Definitive Airway Management

Read through each scenario as a team. Discuss your airway intervention for each scenario. Each scenario requires intervention. For each scenario, what is your airway management?

- Intubate from Above
- Intubate Stoma/Replace Tracheostomy Tube
- Wait for Help to Arrive

##### Scenario #1

60-yo-.M with a history of tracheostomy. Presents with concern for trach obstruction in the setting of respiratory illness. You have placed the patient on oxygen, and the monitor. You have removed the cap, and the inner cannula deflated the cuff and suctioned down the trach. The patient continues to be in respiratory distress and needs definitive airway management.

- Trach > 7 days old
- Trach <7 days old
- Trach <7 days and you have upper airway obstruction

##### Scenario #2

55-yo-F with a history of tracheostomy. Presents with concern for accidental decannulation. You have placed the patient on oxygen and the monitor. You have assessed the stoma. Patient is in respiratory distress and definitive airway management is needed. What are your next steps?

- Trach > 7 days old
- Trach <7 days old
- Trach <7 days and you have upper airway obstruction

#### Group Application Exercise - Activity #4 – Bleeding Tracheostomy

##### Scenario #1

28-yo-male who presents to the ED complaining of bleeding from his tracheostomy. Tracheostomy was placed six weeks ago after motor vehicle accident and traumatic brain injury.

Initial Vital Signs: T 98.6° F, BP 90/60 mmHg, HR 130 bpm, SpO2 85%. You note significant frank blood in and around the tracheostomy.

1. What is the feared complication?
2. Describe the location of the artery in relation to the trachea.

Back to our patient. You note a cuffed tube on examination.

3. What is the next best step in management of the patient?
4. How much air do you use?

After you overinflate the balloon, you note that the bleeding continues.

5. Describe your next step of management.

After completing this step. You prepare to intubate from above. After intubation, you contact ENT for surgical management of patient’s condition.

##### Scenario #2

28-yo-male who presents to the ED complaining of bleeding from his tracheostomy. Tracheostomy was placed six weeks ago after motor vehicle accident and traumatic brain injury.

Initial Vital Signs: T 98.6° F, BP 90/60 mmHg, HR 130 bpm, SpO2 85%. You note significant frank blood in the and around the tracheostomy.

You note an uncuffed tube on examination. You immediately provide compression to the innominate artery using a hooked finger.

1. What is the next best step in management of the patient?

##### Scenario #3

28-yo-male who presents to the ED complaining of bleeding from his tracheostomy. Tracheostomy was placed six weeks ago after motor vehicle accident and traumatic brain injury.

Initial Vital Signs: T 98.6° F, BP 110/60 mmHg, HR 90 bpm, SpO2 95%. You note significant frank blood in and around the tracheostomy.

You note a cuffed tube on examination. You overinflate the balloon and bleeding stops.

1. What is the best imaging of choice to evaluate the patient’s condition?
2. After your imaging results, what is your next step in evaluation of the patient’s condition?

## INSTRUCTOR MATERIALS

Answer keys to all exercises with explanations, are on the following pages.

Learners: Please do not proceed.

### Troubleshooting the Trach TBL: Readiness Assessment Test Key (RAT Key)

1. Which of the following patients can be intubated from above?
  - Laryngectomy Patients
  - Laryngectomy and Tracheostomy Patients
  - **Tracheostomy Patients ***
  - Neither Laryngectomy or Tracheostomy Patients
  **Explanation: Patients who undergo tracheostomy have an intact airway and connection from the oropharynx through the vocal cords to the trachea. This allows them to be intubated via the oropharynx if necessary. Patients who have had a laryngectomy no longer have an intact connection between their oropharynx and trachea. Thus, they cannot be intubated from above.**
2. Which of the following is not a component of a tracheostomy tube?
  - Flange
  - Inner Cannula
  - Obturator
  - Outer Cannula
  - **Regulator***
  **Explanation: There are several parts of tracheostomy equipment that should be readily identified by trainees and physicians. They can be found in the image below. A “Regulator” is not part of tracheostomy equipment. The Flange is the wide portion that sits outside the patient’s skin and prevents the tracheostomy tube from slipping into the airway. This is also where the tracheostomy ties are secured. The inner cannula is a safety feature that serves to prevent mucous build up inside of the tracheostomy tube. The inner cannula provides a lining that is removable and allows for easy cleansing prior to replacement. The obturator is used during tracheostomy placement or tracheostomy exchange. It serves as a guide to ensure appropriate positioning of the tracheostomy tube. The tracheostomy outer cannula serves as the permanent tube that remains in the stoma at all times.**
3. At what postoperative day can a tracheostomy be safely exchanged via the stoma?
  - Post-op Day 1
  - Post-op Day 5
  - Post-op Day 6
  - **Post-op Day 14***
  **Explanation: A tracheostomy stoma is considered to be mature after postoperative day 7. Prior to postoperative day 7, if the tracheostomy is accidentally decannulated, then patient should be intubated via their oropharynx. Immature stomas are at risk for rapid closure, creation of false tracts, and bleeding.**
  Reference: Abouzgheib W, Ross SE. Bedside tracheostomy in the intensive care unit. In: Parrillo JE, Dellinger RP, eds. *Critical Care Medicine: Principles of Diagnosis and Management in the Adult*. Elsevier; 2019:193–200.
4. What is the second step in the algorithm for the treatment of a patient with an obstructed tracheostomy?
  - Attempt to pass a suction catheter
  - Deflate the cuff
  - Intubate from above.
  - Remove any speaking valve or external caps if present
  - **Remove the inner cannula***
  **Explanation: The algorithm for management of a patient with an obstructed tracheostomy can be found referenced below. The initial step is to remove any external devices from tracheostomy that could be contributing to obstruction. For example, these can include speaking valves or external caps. The next step is to remove the inner cannula. If this does not relieve obstruction, then a suction catheter can be passed to attempt to remove any mucous plugs that may be contributing. Next, the cuff should be deflated (if present). Lastly, the tracheostomy should be removed and oropharyngeal intubation pursued.**
  Reference: McGrath BA, Bates L, Atkinson D, Moore JA. Multidisciplinary guidelines for the management of Tracheostomy and laryngectomy airway emergencies. *Anaesthesia*. 2012;67(9):1025–1041. doi:10.1111/j.1365-2044.2012.07217.x
5. A patient presents three days after discharge from prolonged hospital stay for respiratory failure requiring tracheostomy placement with bleeding from his tracheostomy site. His tracheostomy was placed 10 days ago. On inspection, he has a large amount of frank, bright red blood in his tracheostomy. His vital signs are BP 85/55, HR 125, Oxygen saturation 88% on room air, Temp 98.6° F, and RR 32. What is the first step in management of his condition?
  - **Overinflate the tracheostomy cuff** *
  - Remove tracheostomy and intubate from above
  - Go directly to the operating room
  - Remove tracheostomy and apply direct pressure with digital manual compression anteriorly
  **Explanation: This case vignette is describing a patient presenting with tracheoinnominate fistula. Tracheoinnominate fistula (TIF) is a rare but life-threatening complication of tracheostomy placement. TIF typically occurs between three days to three weeks postoperatively and presents with large volume bleeding from the tracheostomy site. The first step in management is to trial overinflation of the cuff to see if this can tamponade the bleeding. The next step is to trial the Utley maneuver by removing the tracheostomy and applying direct pressure via digital compression anteriorly of the innominate artery against the manubrium. Surgical consultation should be obtained immediately for definitive repair. Patients will often require massive transfusion protocol.**
  Reference: Kondajji A, Dombrowska A, Allemang M, Santoscoy T. Emergent management of a tracheoinnominate fistula in the community hospital setting. *Cureus*. Published online June 2, 2020. doi:10.7759/cureus.8403

### Troubleshooting the Trach TBL: Group Application Exercise (GAE) Key

#### Group Application Exercise - Activity #1 – Tracheostomy Basics

1. Identify the parts of the tracheostomy:
2. Your patient needs to be ventilated. Which tracheostomy tube can you attach a bag-valve-mask (BVM) for ventilation?
  **You can attach a BVM to all cuffed tracheostomies. If it is an uncuffed trach, you must replace the trach with a cuffed tube or replace from above.**
3. What part of the tracheostomy tube do you attach to the BVM?
  **You attach a BVM onto the inner cannula for ventilation.**
4. Your patient is unsure of their surgical history. Identify which patient had a tracheostomy and which had a laryngectomy.
  **Tracheostomy patients can be ventilated via their stoma, oropharynx, and nasopharynx. Additionally, you can provide oxygen or BVM via the face or stoma. If you provide ventilations on the face, occlude the stoma with a gauze. Laryngectomy patients no longer have a connection to the pharynx. They are only able to be intubated, ventilated or oxygenated via their stoma.**
5. Your patient is unsure of their type of tracheostomy tube. How can you tell the size of the tube? How can you tell if it is cuffed or uncuffed?
  **The face plate or flange of the tube will have the size and brand listed. You can tell if it is cuffed or uncuffed based on if there is a pilot balloon. Typically, on the pilot balloon will be stated how much air the cuff can hold.**
6. Your patient has a 6.0 cuffed Shley tube. You do not have this item stocked in your outlying hospital. What size endotracheal tube (ETT) could you place in the stoma?
  **Trach sizing is the same as ETT. So, a 6.0 cuffed tracheostomy tube would be able to take a 6.0 ETT.**

#### Group Application Exercise - Activity #2 – Airway Algorithm

You are working in a rural emergency department (ED) as a single coverage physician when you receive a patient from a nearby skilled nursing facility for respiratory distress. The patient has a history of anoxic brain injury and has had a tracheostomy for many years. EMS state they were called by the nursing facility staff for low oxygen saturation and difficulty breathing.

Initial Vital Signs: HR 120 bpm, RR 36, BP 105/66, O2 72% on room air

They placed a non-rebreather mask on the patient and quickly transported him to you.

1. What is your first step in evaluating this patient?
  **Place patient on monitors. Maintain supplemental oxygen via mask to both face and tracheostomy site. Is the patient spontaneously breathing, and do they have a pulse? Identify the trach equipment.**
2. What questions are important for you to ask this patient?
  **It is important to ask the following questions:**
  **3 Whats?**
  - **What kind of trach? What size?**
  - **Cuffed or Uncuffed?**
  - **What other trach complications?**
  **2 Whens?**
  - **When was the tracheostomy tube placed?**
  - **If decannulated, when did it come out?**
  **1 Whys?**
  - **Why did they place a trach?**

The patient has thready, rapid peripheral pulses. He is breathing spontaneously and appears to be in severe respiratory distress. His O2 on the monitor is 80% on NRB over the mouth. You assess his tracheostomy equipment and see a purple cap covering the inner cannula. You suspect airway obstruction.

3. What is the step that needs to be taken to relieve the airway obstruction?
  **The first step in management of this patient is to remove the speaking valve and evaluate if this maneuver relieves respiratory distress and obstruction.**

Once you have removed the speaking cap, you examine the equipment below.

4. What is the next step in attempting to relieve airway obstruction? Hint: this step relieves the majority of airway obstructions.
  **The next step is to remove the inner cannula of the tracheostomy to see if this relieves obstruction.**

The patient’s oxygen saturation improves minimally to 86%, but he still appears to have some increased work of breathing with coarse breath sounds and stridor.

5. Describe the steps of how to set up suctioning for a tracheostomy patient.
  **The next step in the algorithm for management of airway obstruction in a patient with a tracheostomy is to attempt to pass a catheter and suction the patient. Suctioning can assist with removal of mucus plugs that can be totally or partially obstructing the patient’s airway.**
  **Steps for Suctioning:**
  1. **Connect suction tubing to wall suction and turn wall suction to continuous.**
  2. **Connect tracheostomy suction tubing (as shown above) to suction catheter.**
  3. **Advance tubing through stoma until resistance is met with suction control vent uncovered.**
  4. **Slightly retract tubing then place thumb over suction control vent to initiate suction of airway.**
  5. **Gently twist/turn the suction catheter while removing it during suction. Don’t suction for longer than ten seconds. Do not suction except when actively withdrawing the catheter.**
  6. **Repeat as necessary.**

You attempt to perform suctioning of the patient but are unable to pass the suction catheter through the stoma. The patient remains hypoxic, tachypneic, and with stridor. You attempt to bag the patient via their tracheostomy tube; you are unable to get adequate tidal volumes and bagging is extremely difficult.

You follow the next step in the algorithm to aid the patient’s obstruction. You deflate the cuff and see if it helps the obstruction.

6. How can you determine how much air should be removed from the patient’s tracheostomy balloon to adequately deflate the cuff?
  **The amount of air that can be held in the cuff should be listed on the face plate or pilot balloon. It is important to quickly assess the patient’s equipment and identify the type of tracheostomy, size of tracheostomy, and volume of balloon cuff if present.**

You deflate the cuff and the patient’s respiratory distress subsides. His oxygen saturation returns to normal. Your respiratory therapist assists with applying humidified oxygen and eventually you are able to suction out large amounts of mucus. He is found to have atelectasis on his chest radiograph likely from mucous plugging. You admit for observation.

#### Group Application Exercise - Activity #3 - Definitive Airway Management

Read through each scenario as a team. Discuss your airway intervention for each scenario. Each scenario requires intervention. For each scenario, what is your airway management?

d. Intubate from Above
e. Intubate Stoma/Replace Tracheostomy Tube
f. Wait for Help to Arrive

##### Scenario #1

60-yo-.M with a history of tracheostomy. Presents with concern for trach obstruction in the setting of respiratory illness. You have placed the patient on oxygen, and the monitor. You have removed the cap, and the inner cannula deflated the cuff and suctioned down the trach. The patient continues to be in respiratory distress and needs definitive airway management.

- Trach > 7 days old
  1. **Remove and replace the tracheostomy.**
  2. **Can use the same size endotracheal tube (ETT) if unable to find a tracheostomy tube of an appropriate size. Insert only 1–1.5 cm past the cuff.**
  3. Trach <7 days old
  4. **Intubate from above as your normal practice.**
  5. **Cover the stoma with nonocclusive dressing to maintain seal.**
  6. Trach <7 days and you have upper airway obstruction
  7. **Prepare for difficult intubation, page Anesthesia/ENT. Obtain a fiberoptic scope.**
  8. **Remove tracheostomy tube. (Remember the tube is obstructed. It is not a patent airway if obstructed.)**
  9. **Use a pediatric BVM or laryngeal mask airway (LMA) to create a seal over stoma.**
  10. **Consider using a scope to enter the stoma for intubation if Anesthesia/ENT is unavailable.**

##### Scenario #2

55-yo-F with a history of tracheostomy. Presents with concern for accidental decannulation. You have placed the patient on oxygen and the monitor. You have assessed the stoma. Patient is in respiratory distress and definitive airway management is needed. What are your next steps?

- Trach > 7 days old
  1. **Remove and replace the tracheostomy.**
  2. **Can use the same size endotracheal tube (ETT) if unable to find a tracheostomy tube of an appropriate size. Insert only 1–1.5 cm past the stoma.**
  3. Trach <7 days old
  4. **Intubate from above as your normal practice.**
  5. **Cover the stoma with nonocclusive dressing to maintain seal.**
  6. Trach <7 days and you have upper airway obstruction
  7. **Prepare for difficult intubation, page Anesthesia/ENT. Obtain a fiberoptic scope.**
  8. **Remove tracheostomy tube.**
  9. **Use a pediatric BVM or LMA to create a seal over stoma.**
  10. **Consider using a scope to enter the stoma for intubation if Anesthesia/ENT is unavailable.**

#### Group Application Exercise - Activity #4 – Bleeding Tracheostomy

##### Scenario #1

28-yo-male who presents to the ED complaining of bleeding from his tracheostomy. Tracheostomy was placed six weeks ago after motor vehicle accident and traumatic brain injury.

Initial Vital Signs: T 98.6° F, BP 90/60 mmHg, HR 130 bpm, SpO2 85%. You note significant frank blood in and around the tracheostomy.

1. What is the feared complication?
  **Concerning complication is the development of a tracheoinnominate fistula (TIF).**
2. Describe the location of the artery in relation to the trachea.
  **Pressure from tracheostomy tip or cuff erodes into the innominate artery that lies anterior to the trachea.**

Back to our patient. You note a cuffed tube on examination.

3. What is the next best step in management of the patient?
  **Overinflate the tracheostomy cuff.**
4. How much air do you use?
  **Ensure the pilot balloon is fully inflated. Once fully inflated, add 5cc of air to the cuff to balloon.**

After you overinflate the balloon, you note that the bleeding continues.

5. Describe your next step of management.
  **If there is still bleeding, the site is likely distal to the tip of the current tube. Insert a cuffed endotracheal tube (either by intubating orally or through the stoma) deep to the bleeding. Then insert one finger into the stoma and apply pressure to the innominate artery. If you cannot reach the artery with your finger, use your thumb in the sternal notch as external pressure and pinch the innominate artery between your two fingers. Maintain this pressure while transporting to the operating room.**

After completing this step. You prepare to intubate from above. After intubation, you contact ENT for surgical management of patient’s condition.

##### Scenario #2

28-yo-male who presents to the ED complaining of bleeding from his tracheostomy. Tracheostomy was placed six weeks ago after motor vehicle accident and traumatic brain injury.

Initial Vital Signs: T 98.6° F, BP 90/60 mmHg, HR 130 bpm, SpO2 85%. You note significant frank blood in the and around the tracheostomy.

You note an uncuffed tube on examination. You immediately provide compression to the innominate artery using a hooked finger.

1. What is the next best step in management of the patient?
  **Prepare to intubate from above.**

##### Scenario #3

28-yo-male who presents to the ED complaining of bleeding from his tracheostomy. Tracheostomy was placed six weeks ago after motor vehicle accident and traumatic brain injury.

Initial Vital Signs: T 98.6° F, BP 110/60 mmHg, HR 90 bpm, SpO2 95%. You note significant frank blood in and around the tracheostomy.

You note a cuffed tube on examination. You overinflate the balloon and bleeding stops.

1. What is the best imaging of choice to evaluate the patient’s condition?
  **If the patient is stable, CT angiography is needed to make the diagnosis.**
2. After your imaging results, what is your next step in evaluation of the patient’s condition?
  **All patients should be seen by ENT or the surgical service that placed the tracheostomy should be contacted for management. The patients typically need to be admitted for observation unless another reasonable diagnosis for the bleeding has been identified (ie. granulation tissue).**
